# Case of a Boy Poisoned by the Root of Hemlock-Dropwort

**Published:** 1781-07

**Authors:** 


### SECTION II.

### Essays and Observations.

I. *Cafe of a boy poifoned by the root of hemlock-dropwort. By* Thomas Houlfton, M. D. *Phyfician to the Liverpool Infirmary. Communicated by* William Hawes, M. D. *Phyfician in London. Read* June 25, 1781.

JUNE 9th 1781, the eldeft fon of the Rev. Mr. Kirkpatrick, a diffenting minifter, about nine years old, rambling with feveral other children children in the fields adjoining to the Leeds Canal near Liverpool, gathered, and gave to the others, a number of the roots of the hemlock-dropwort, which he believed were ground nuts, and of which he eat a much greater quantity than the reſt. As he was returning home he grew giddy, and if he had not been prevented would have reeled into the canal. His inability to direct his motions increaſed gradually, and he was ſoon affected with ſtupor and convulſions. His mother, apprized of his ſituation, ſpeedily came to him, and immediately, as ſhe ſaid, conceived the idea of his having eat ſomething, the effects of which were ſimilar to the poiſon adminiſtered to Sir Theodoſius Boughton, till which time no ſuch thing had been apprehended. Some water out of the canal was given him to drink, and he vomited a conſiderable quantity of the root he had ſwallowed; he however grew worſe, raved, became heavy and convulſed, and was carried into a houſe adjoining. Mr. Shertcliffe, a ſurgeon in the neighbourhood, was ſent for, who, with a view to evacuate what he had taken, gave him a ſolution of emetic tartar, and a purgative glyſter.

He had ſwallowed at leaſt twenty grains of tartar emetic, when I was ſent for to him about

 eight eight in the evening. I found him quite in the epileptic ftate, with the pupil vaftly dilated, total infenfibility, and all the appearance of a perfon in the laft degree of intoxication. Convinced that unlefs the contents of the ftomach could be expelled, no hope of his recovery remained, I gave, in folution, a fcruple of white vitriol, moft part of which was got down.

The convulfions, for fome time paft, had been ftrong and frequent; they feemed to begin with an effort, as it were to vomit (though after he got into the houfe he never vomited in the leaft). The head was drawn to the right fide, and thrown back, general fpafms fucceeded, the eyes ftarted prodigioufly out from the fockets, and the tongue was thruft out, and forcibly bit. Some æther was fent for, and I poured a fmall quantity into the mouth, on the temples, &c. It was thought at times to relieve the fits which interrupted the circulation fo as to render the pulfe imperceptible; and to give often reafon to fuppofe it was irrecoverably ftopped. In this manner, however, the fcene was clofed at laft, rather placidly, about ten o'clock at night, after he had fuffered thus above four hours. The refpiration, though flow, continued tolerably eafy, almoft to the the laſt. The clyſter operated a little before he died, and a very offenſive ſtool followed.

Notwithſtanding the boy had thrown up a conſiderable quantity of the root, yet I had no doubt but that ſuch a part of what he had eaten remained in the ſtomach as would render every effort to ſave him ineffectual. The event unfortunately anſwered my expectations, and diſſection confirmed the truth of the conjecture. Mr. Shertcliffe found in the ſtomach above an handful of the root, and noticed very ſenſibly the ſmell peculiar to it, the moment he cut into the cellular membrane, though it was not till twenty-four hours after death.

It was at firſt ſuppoſed that what the boys had gathered and eaten was the water-parſnip; and afterwards that it was the water-hemlock. Indeed Boerhaave, in his *Hiſtoria Plantarum*, under the article *Sium (water-parſnip)* commends the firſt ſpecies for its aperient, emollient, and detergent qualities; but adds, " that he never had dared to adminiſter it, from the reſemblance which it bears to the ſecond ſpecies, the *cicuta aquatica*, of which thoſe who have eaten, unleſs relieved by vomiting, died dreadfully and ſingularly convulſed." The latter (the *water-hemlock)* which is extremely poiſonous, is frequently confounded alſo with the *hemlock-dropwort*, the plant now ſpoken of, which is equally dangerous, and is termed by Lobel, Ray, and others, *Oenanthe cicutæ facie*. This, however it is certain, was the one pitched upon by the boy, who with difficulty recovered, as the root he and his companions had eat of.

Four of the other boys in company had partaken, though more ſparingly, of the noxious repaſt, but on the firſt alarm, vomits having been exhibited, they all eſcaped. One, however, was with difficulty made to vomit, though he took largely both emetic tartar and ipecacoanha, and he was affected with giddineſs, drowſineſs, and twitching ſo much that for ſome hours his recovery remained doubtful. He told me he had eaten one root and a half; and more than two hours had elapſed before he was ſenſibly affected by it.

This unfortunate accident, as well as the one which was lately the ſubject of a judicial diſcuſſion, proves how fatally certain is the effect of the poiſons of this claſs. Theſe vegetable poiſons do not, like the mineral ones, become fatal by producing inflammation of the ſtomach, though at firſt they ſtimulate, and endeavour to promote their own diſcharge, yet their baneful action is

ſolely folely on the nervous fyftem. Like to *opium* or *fpirits*, they bring on fuch a degree of infenfibility, or, as fome fuppofe, of fpafm, as wholly to deftroy or counteract *the power of the ftomach to expel them*, whilft their continuance there muft inevitably prove fatal; whereas many *mineral poifons* may be decompofed by any alkali; and even the danger from drinking *fpirits*, may be greatly leffened, by conveying into the ftomach (by means of a pipe paffed beyond the glottis) large quantities of water to dilute them after the power of vomiting, as well as of fwallowing, is loft. [See two papers which I drew up on this fubject, and which are inferted in the Edinburgh Medical Commentaries, Vol. VI. p. 325, and in thofe by Dr. Duncan, Part 3, 1780.]

To render a poifonous vegetable in the ftomach, which cannot be evacuated, inactive, is what we are yet unequal to.—To dilute it would probably be, at leaft, a vain attempt, if it did not (by the liquid acting as a menftruum) elicit and render more active the poifonous quality; and unfortunately, to evacuate it, after it has remained long enough to produce in a certain degree, its effect on the ftomach, feems next to impoffible. We fhould, however, when there is the eaft ground to fufpect any thing of this kind, immediately immediately endeavour, by an active emetic, to evacuate the ſtomach, whilſt there yet remains a poſſibility of doing it. On the early exhibition of a vomit in ſuch caſes depends its operation, and on that only, perhaps, the ſecurity of the patient.

*Liverpool*, June 15, 1781.

